# Comprehensive analysis of transcriptome and metabolome analysis in Intrahepatic Cholangiocarcinoma and Hepatocellular Carcinoma

**DOI:** 10.1038/srep16294

**Published:** 2015-11-05

**Authors:** Yoshiki Murakami, Shoji Kubo, Akihiro Tamori, Saori Itami, Etsushi Kawamura, Keiko Iwaisako, Kazuo Ikeda, Norifumi Kawada, Takahiro Ochiya, Y-h Taguchi

**Affiliations:** 1 Department of Hepatology, Graduate School of Medicine, Osaka City University, 1-4-3 Asahimachi, Abeno-ku, Osaka 545-8585, Japan; 2 Department of Hepato-Biliary-Pancreatic Surgery, Graduate School of Medicine, Osaka City University, 1-4-3, Asahimachi, Abeno-ku, Osaka 545-8585, Japan; 3 Department of Target Therapy Oncology, Kyoto University Graduate School of Medicine, 54 Shogoin Kawahara-cho, Sakyo-ku, Kyoto 606-8507, Japan; 4 Department of Anatomy and Regenerative Biology, Graduate School of Medicine, Osaka City University, 1-4-3, Asahimachi, Abeno-ku, Osaka 545-8585, Japan; 5 Division of Molecular and Cellular Medicine, National Cancer Center Research Institute, 5-1-1 Tsukiji, Chuo-ku, Tokyo 104-0045, Japan; 6 Department of Physics, Chuo University, 1-13-27 Kasuga, Bunkyo-ku, Tokyo 112-0003, Japan

## Abstract

Intrahepatic cholangiocarcinoma (ICC) and hepatocellular carcinoma (HCC) are liver originated malignant tumors. Of the two, ICC has the worse prognosis because it has no reliable diagnostic markers and its carcinogenic mechanism is not fully understood. The aim of this study was to integrate metabolomics and transcriptomics datasets to identify variances if any in the carcinogenic mechanism of ICC and HCC. Ten ICC and 6 HCC who were resected surgically, were enrolled. miRNA and mRNA expression analysis were performed by microarray on ICC and HCC and their corresponding non-tumor tissues (ICC_NT and HCC_NT). Compound analysis was performed using capillary electrophoresis time-of-flight mass spectrometry (CE-TOFMS). Principle component analysis (PCA) revealed that among the four sample groups (ICC, ICC_NT, HCC, and HCC_NT) there were 14 compounds, 62 mRNAs and 17 miRNAs with two distinct patterns: tumor and non-tumor, and ICC and non-ICC. We accurately (84.38%) distinguished ICC by the distinct pattern of its compounds. Pathway analysis using transcriptome and metabolome showed that several pathways varied between tumor and non-tumor samples. Based on the results of the PCA, we believe that ICC and HCC have different carcinogenic mechanism therefore knowing the specific profile of genes and compounds can be useful in diagnosing ICC.

Intrahepatic cholangiocarcinoma (ICC) is the second most common hepatic cancer and accounts for 10–25% of all hepatic malignant tumors^1^,^2^. Hepatocellular carcinoma (HCC) represents the major histological subtype of primary liver malignancies, accounting for 70% to 85% of the total liver cancer burden^3^. Most cases of HCC (75% to 90%) are found in patients with liver cirrhosis resulting from chronic hepatitis B or C infection, alcoholic injury, and recently in non-alcoholic steatohepatitis (NASH)^4^,^5^. ICC is labeled as a malignant tumor arising from the peripheral intrahepatic bile duct epithelium^1^. High risk factors associated with ICC includes being male^6^ being over age 65^7^, and having primary sclerosing cholangitis (PSC)^8^, biliary duct cysts^9^, hepatolithiasis^10^, and chemical toxin overload^11^. It was also previously reported that genetically impaired biliary excretion of phospholipids is an underlying mechanism of ICC^12^,^13^. Metabolomic investigations support this view, as lower phosphatidylcholine and elevated glycine- and taurine-conjugated bile acids have been reported in the bile of ICC patients^14^,^15^.

Several pathways and genetic alternations have been associated with ICC development. For example, aberrant glucose and lipid metabolism as well as 15-hydroxyprostaglandin dehydrogenase-mediated 15-keto-prostaglandin E2 signaling cascade inhibited ICC cell growth via peroxisome proliferator-activated receptor-gamma, Smad2/3, and TAP63 pathway^16^. The endocannabinoid anandamide exerts an anti-proliferative effect on ICC through one of the cannabinoid G-protein coupled receptor 55, and by stabilizing lipid rafts. This allows for the recruitment and activation of the Fas receptor complex^17^. Simvastatin is known to induce ICC cell death by disrupting Rac1/lipid raft co-localization and by depressing Rac1 activity^18^. Aberrant miRNA expression has also been associated with ICC; notably, miR-21 has been implicated in cell proliferation, apoptosis, metastasis, and migration^19^,^20^. let-7a was also found to be up-regulated in ICC and contributes to the survival of cholangiocytes via enforced IL-6 activity^21^,^22^.

ICC is characterized by histological observations of dissected tumors. It is difficult to conclude whether ICC is truly derived from cholangiocytes as it was reported that patients with hepatitis C virus infection often develop ICC, suggesting that ICC is derived from transformed hepatocytes^23^. Several reports have suggested that Notch activation is critical for hepatocytes to convert into biliary lineage cells during the onset of ICC and its subsequent malignancy and progression. In these said studies therefore, ICC was generated by biliary lineage cells derived from hepatocytes, rather than from cholangiocytes^24^. ICC is known to have a poorer survival outcome than HCC mainly due to the advanced tumor stage of patients with intrahepatic metastasis at presentation and early postoperative recurrence^25^. New biomarkers that can detect ICC, especially in its early stages, and that can help clarify the mechanism of ICC are needed to increase the survival rate for ICC patients.

In this study, we performed a comprehensive analysis using transcriptome and metabolome to discover (1) if there is a difference in the carcinogenic process between ICC and HCC, and (2) accurate and sensitive molecular markers to diagnose ICC.

## Result

### Variable selection using principal component

**Figure 1:**
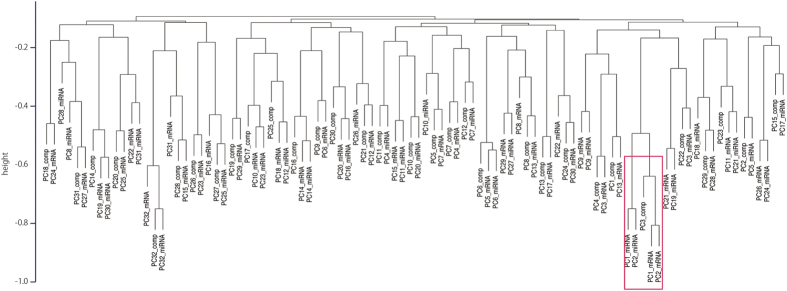
Hierarchical Clustering using correlation coefficients. Hierarchical clustering of 96 PCs: consisting of 32 PCs each obtained from mRNAs, miRNAs and compounds. Each PC consists of 32 dimensional vectors with 32 elements, each of which corresponds to the contribution of each sample to each PC. The correlation coefficients between PCs were computed using these 32 elements. On the Vertical axis are absolute negative correlation coefficients that are used as distance for hierarchical clustering (lower pairs have larger absolute correlations). Red rectangle indicates 5PCs which were chosen by hierarchical clustering.

Each mRNA, miRNA or compound taken from the thirty two samples (10 pairs of ICC and ICC-NT, and 6 pairs of HCC and HCC-NT) (Supplementary Table 1), was considered as a point in a 32 dimensional space and embedded in a low dimensional space using principal component analysis (PCA). Figure 1 shows the hierarchical clustering of the principal components (PCs). The vertical axis exhibits the negative correlation coefficients used to define the distance measures of the clusters. Since each CX_j_^k^ (j,k = 1, 2,,, 32) is a composite of the 32 samples, CX^k^ = (CX_1_^k^,CX_2_^k^,…,CX_32_^k^) was also expressed as 32-dimensional vectors (see supplementary method). To reiterate, the primary aim of this study was to perform an integrated analysis of compounds, mRNA, and miRNA, to identify those that are related to ICC and HCC. The numbers attached to PCs represent the order of PCs while smaller numbers indicate larger contributions to overall variances. Using Unweighted Pair Group Method with Arithmetic Mean (UPGMA) we performed separate PCA for compounds, mRNA and miRNA. The PCs were chosen based on the following two criteria: (1) The PC cluster should be located in the lower right position, in other words, the absolute value of the correlation coefficient should be the largest. (2) k of selected CX^k^ should be as small as possible, since a smaller k indicates more contributions. Five PC (loading)s fulfilled these criteria: PC3_comp (PC3_ compound), PC1_mRNA, PC2_mRNA, PC1_miRNA and PC2_miRNA (Fig. 3).

**Figure 2:**
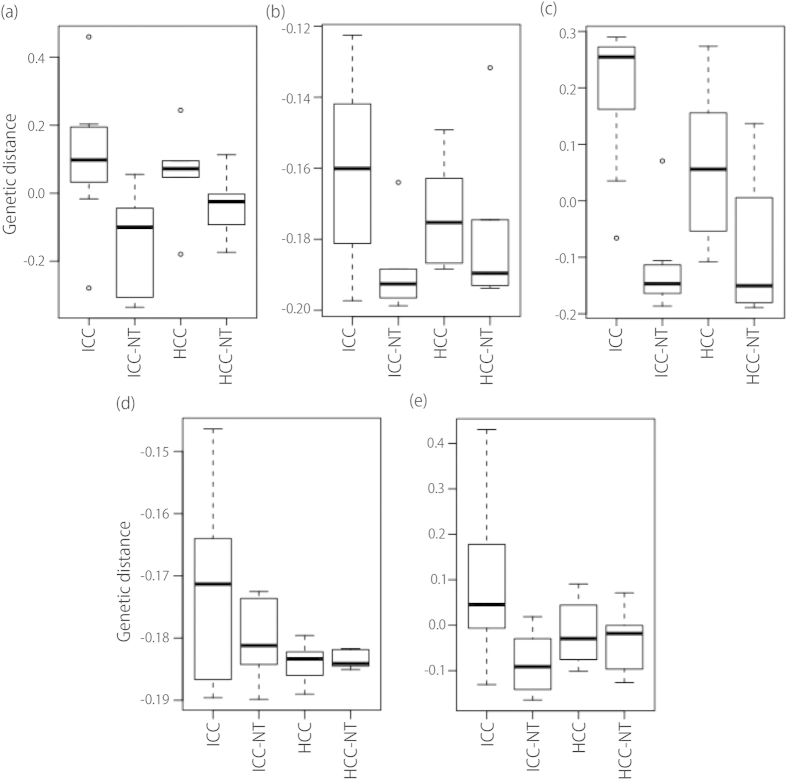
Scatter diagram. Lower triangle: Scatter plot between five PCs (**a**) PC3_comp, (**b**) PC1_mRNA, (**c**) PC2_mRNA, (**d**) PC1_miRNA, and (**e**) PC2_miRNA, selected based on the hierarchical clustering shown in Fig. 1. Each row and column corresponds to the PC displayed diagonally. Open rectangle in the second row and third column indicates the correlation coefficients (upper numerical value) and their P-values (lower numerical value) associated with the corresponding scatter plots.

In order to validate the correlation of each selected PC, we performed a scatter plot analysis (Fig. 2). A comparison was done of the expression patterns of compound, miRNA, and mRNA among ICC, ICC-NT, HCC, and HCC-NT. As expected ICC and HCC had expressions that were markedly distinct from their non-tumor (NT) counterpart; however, the difference between ICC and ICC-NT was greater than HCC and HCC-NT (Fig. 2). Each row and column corresponds to the PCs displayed diagonally. For example, the scatter plot in the third row and second column is between PC1_mRNA and PC2_mRNA. Open rectangles in the second row and third column indicate correlation coefficients (upper numerical value) and their P-values (lower numerical value) associated with the corresponding scatter plots.

Next, to quantitatively confirm that selected PCs were significantly distinct among HCC, ICC, HCC-NT and ICC-NT, we performed the following categorical regression analysis.


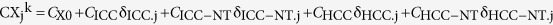


**Figure 3:**
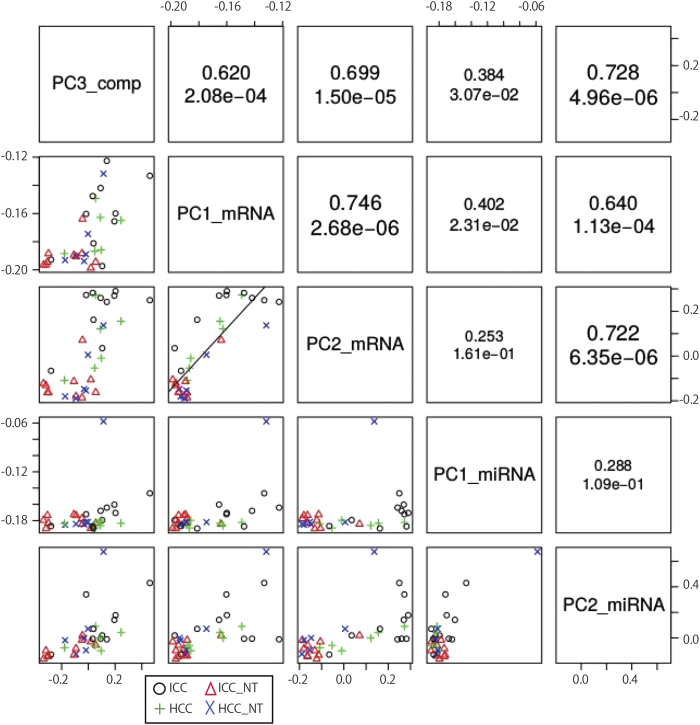
Box plot in 5 PCA. Box Plot of PCs is shown in Fig. 2. The sample contribution to each PC are shown using boxplot classified based on four sample classes. The correlation coefficients used for the hierarchical clustering tree represented in Fig. 1 and shown in the upper triangle in Fig. 2 are for the distances found among the five plots. Vertical line represents genetic distance. Red dots are depicted as measured value of each probe after normalization. P values are shown in a scatter plot (P value of PC3_comp, PC1_mRNA, PC2_mRNA, PC1_miRNA, and PC2_miRNA, correspond to 8.68e-03, 1.69e-02, 3.98e-06, 4.74e-02, and 9.42e-03, respectively).

Here δ_a,i_ takes one if the i-th sample is equivalent to category a (a represents ICC, ICC-NT, HCC, or HCC-NT), otherwise it had a value of zero. CX_i_^k^ reflects the contribution of the sample i to the kth principal component. We employed categorical regression instead of ordinary multivariate analysis because the order and magnitude among the four groups (ICC, ICC/-NT, HCC, and HCC-NT) was unknown. The genetic distance between ICC and ICC-NT appeared larger than between HCC and HCC-NT (Fig 3). All together, this data suggests that we successfully selected PCs that accurately distinguished two distinct patterns (tumor/non-tumor and ICC/non-ICC) among the four groups. More detailed methodological and theoretical background can be found in our previous publications^26^,^27^ and supplementary methods.

### Selection of ICC related compound, mRNA, and miRNA

**Figure 4:**
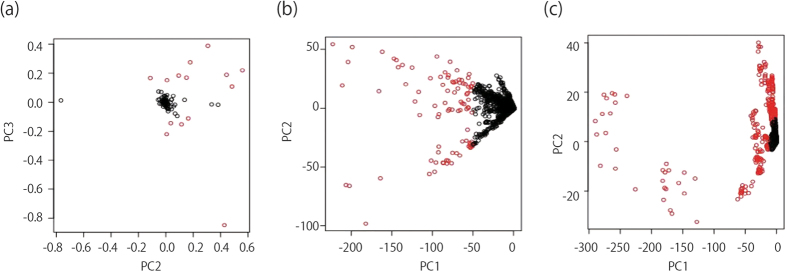
Embedding of compound, mRNA, and miRNA. (**a**) Two-dimensional embedding of compounds spanned by PC2 and PC3. (**b**) Two-dimensional embedding of mRNA spanned by PC1 and PC2. (**c**) Two dimensional embedding of miRNA spanned by PC1 and PC2. We chose compounds, mRNA and miRNAs as “outliers” for further analysis.

We used PCA based unsupervised FE to select the ICC related compounds, mRNA, and miRNA^28^. Results from scatter plots in five PCs, showed that each of the four groups had compound levels or gene expression patterns that were distinct. Figure 4 shows two-dimensional embedding of mRNA/miRNA spanned by PCXi1 and PCXi2 and compounds spanned by PCX_i_2 and PCX_i_^3 all^ obtained by PCA (see supplementary method). Each circle in Fig. 4 corresponds to an individual compound detected by CE-TOFMS or individual mRNA/miRNA expression (probe) on microarray plate. Red circles are “outliers” selected for further analysis: compounds along PC3, mRNA along PC1 and miRNAs along PC1 and PC2. In total 14 outlying compounds, 62 mRNAs, and 17 miRNAs, were selected since they had larger contributions towards the 5PCs (red circle in figure selection) (Fig. 4), (Supplementary Table 2, 3, 4).

**Figure 5:**
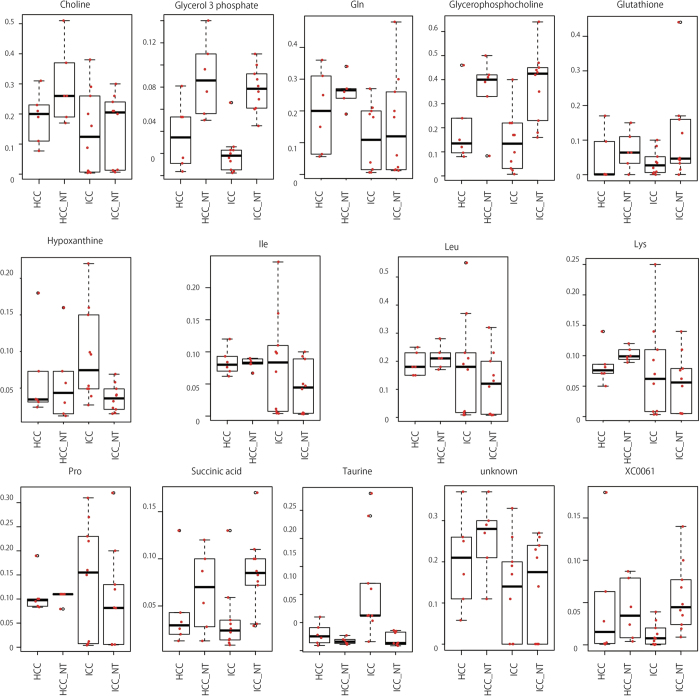
Box plot of selected compounds. Amount of 14 compounds used to discriminate between tumor (ICC and HCC) and non-tumor (ICC_NT and HCC_NT) or between ICC and non-ICC (ICC-NT, HCC, and HCC-NT). Left vertical axis shows the amount of compound. Red dots are depicted as measured value of each probe after normalization. Each p-value is indicated in Table S2. XC00061 is only known as Pubchem accession number (http://www.ncbi.nlm.nih.gov/pccompound). Unknown: neither the name of component nor the Pubchem accession number is known.

Fourteen compounds (Fig. 5 and Supplementary Table 2), were selected that could separately distinguish each of our four sample groups. For example, Glycerol 3−phosphate, Succinic acid and Glycerophosphocholine were differentially expressed in the tumor (HCC and ICC) and non-tumor (HCC-NT and ICC-NT) population. A distinction between tumor and non-tumor samples was also seen in PC3 of compound (Fig. 3). Thus we surmise that it is due to these three compounds that a distinction can be made between tumors and non-tumors. On the other hand, four amino acids (Lys, Pro, Leu and IIe) were more diversely expressed in ICC/ICC-NT than in HCC and HCC-NT. Since this was also observed in PC3 (Fig. 3), we believe these four amino compounds are primarily responsible for ICC/ICC-NT having a larger genetic distance than HCC and HCC-NT. Hypoxanthine and Taurine were the only compounds that had distinct expressions that differentiated ICC from HCC, HCC-NT and ICC-NT which suggests that these two compounds allowed ICC to be distinguished from other tumor and non-tumor samples.

**Figure 6:**
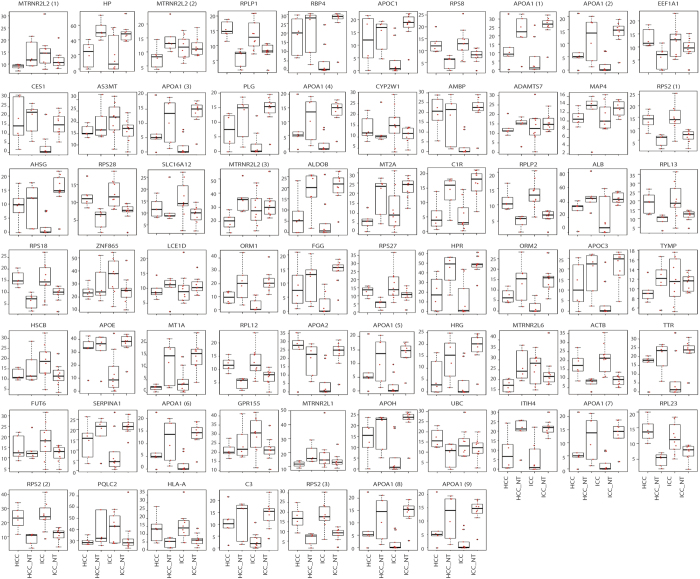
Box plot of selected mRNAs. Expression pattern of 62 mRNA used to discriminate between tumor (ICC and HCC) and non-tumor (ICC_NT and HCC_NT) or between ICC and non-ICC (ICC-NT, HCC, and HCC-NT). In cases where there are multiple probes for one mRNA, pleural box plots with the same mRNA are listed. Vertical left axis shows the expression level of mRNAs. Red dots are depicted as measured value of each probe after normalization. P-values are indicated in Table S3.

Multiple probes can be mapped to a singular gene in order to increase sensitivity and specificity; several mRNAs (APOA1, MTRNR2L2, and RPS2) in this study had multiple probes resulting in a total of 67 probes for 62 mRNAs. Abbreviations for genes are shown in Table S3. Among these 62 mRNAs (Fig. 6 and Supplementary Table 3), there were several groups that share similar expression with compounds. For example, HRP, HP, APOA1, ALDOB, ITIH4, ORM1, SERPINA1, HRG, and MT2A are mRNAs that express differentially between tumors (HCC and ICC) and non-tumors (ICC-NT and HCC-NT), while ALB, APOE, RBP4, TTR, AMBP, APOA2, APOC3, APOH, CES1 and APOC1 are expressed in ICC distinctly from the other three samples. We observed two varying patterns among mRNA: one is the distinction between tumors and non-tumors reflected by PC2_mRNA; the other is the distinction between ICC and the other three groups, which are reflected by PC1_mRNA (Fig. 3).

**Figure 7:**
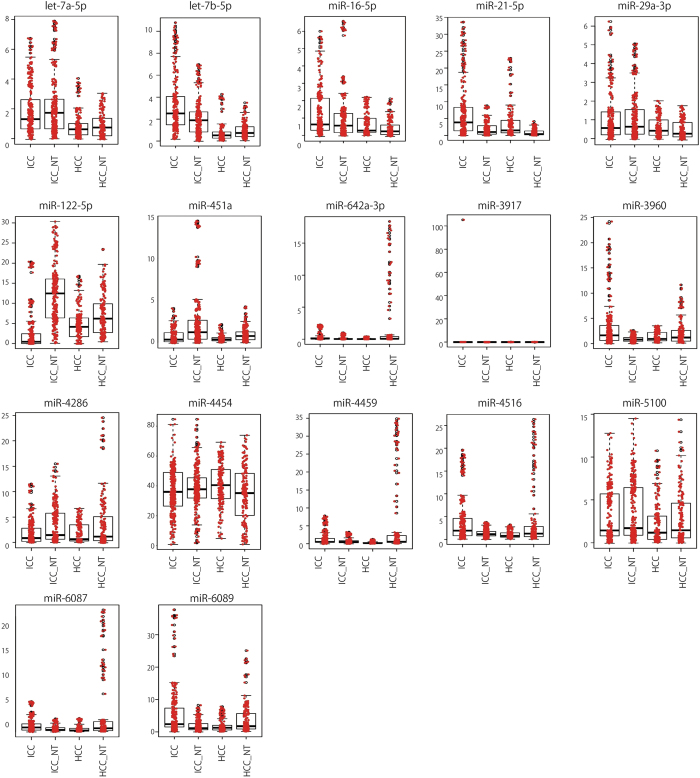
Box plot of selected miRNAs. Expression pattern of 17 miRNAs used to discriminate between tumor (ICC and HCC) and non-tumor (ICC_NT and HCC_NT) or between ICC and non-ICC (ICC-NT, HCC, and HCC-NT). Vertical left axis shows miRNA expression levels. P-values are indicated in Table S4.

Similarly, two clear distinctions were observed between tumors and non-tumors, and ICC and non-ICC among 17 miRNAs (Fig. 7 and Table S4). The former group consisted of miR-21-5p, miR-122-5p, miR-451a, and miR-4286, while let-7b-5p and miR-16-5p fell in the latter. These two tendencies were also observed in PC2 and PC1 in miRNAs (Fig. 3).

### ICC related carcinogenetic pathway

In order to clarify the biological significance of the selected compounds and genes Integrated Molecular Pathway Level Analysis (IMPaLA) (http://impala.molgen.mpg.de) was used to calculate the pathway enrichment of the compounds and mRNA chosen from pathways, such as KEGG and REACTOME as well as other data sets. The list of pathways with P values for which multiple comparisons and adjustments were carried out is shown (p < 0.05) in supplementary Table 5 and 6.

In the heatmap in Supplementary Fig. 1 and 2 the black boxes represent either compound or mRNAs. Similar pathways were classified under one color cluster using UPGMA. For example, C00819 (Glutamine) contributes to D-glutamine and D-glutamate metabolism. The largest cluster (red) represents pathways related to tRNA synthesis (tRNA charging, tRNA aminoacylation, Cytosolic tRNA aminoacylation, Mitochondrial tRNA amicoacylation, Aminoacyl-tRNA biosynthesis), and amino acid synthesis (glutathione synthesis and recycling, amino acid transport across the plasma membrane, amino acid and oligopeptide SLC transporters, transport of inorganic cations/anions and amino acids/oligopeptide). The green cluster shows lipoprotein related pathways: metabolism of lipids and lipoproteins, acetylcholine Synthesis, glycerophospholipid metabolism, phospholipid metabolism, and hydrolysis of LPC (Supplementary Fig. 1).

In the mRNA analysis performed using DIANA-miRPath (http://diana.imis.athena-innovation.gr/DianaTools/index.php?r=mirpath/index), the yellow cluster represents the lipoprotein related pathways (PPAR signaling pathway, HDL−mediated lipid transport, lipoprotein metabolism, lipid digestion_ mobilization_ and transport, statin pathway, chylomicron−mediated lipid transport, scavenging of heme from plasma, binding and uptake of ligands by scavenger receptors, FOXA2 and FOXA3 transcription factor networks). The red cluster shows the transcription related pathways including the tRNA relation pathway (activation of the mRNA upon cap−binding, ribosomal scanning and start codon recognition, peptide chain elongation, eukaryotic translation elongation, metabolism of RNA, metabolism of mRNA, ribosome, eukaryotic translation initiation, cap−dependent translation initiation, SRP−dependent cotranslational protein targeting to GTP hydrolysis and joining of the 60S ribosomal subunit, L13a−mediated translational silencing of ceruloplasmin 3−UTR−mediated translational regulation) (Supplementary Fig. 2).

Pathway analysis of the validated miRNAs using DIANA-miRPath pointed to 62 cancer-related pathways (p < 0.05). RNA synthesis related pathway (RNA transport, Ribosome biogenesis in eukaryotes, Ribosome, RNA polymerase, Aminoacyl-tRNA biosynthesis) and glucose-lipid synthesis pathway (Adipocytokine signaling pathway, Insulin signaling pathway) were found to be associated with ICC carcinogenesis (Supplementary Table 7).

### Diagnosing ICC using compounds and miRNA expression pattern

We attempted to identify diagnostic biomarkers for ICC. We used leave one out cross validation (LOOCV) to classify samples into three groups: ICC, HCC, and non-tumorous. Using the profile of 14 compounds (Supplementary Table 2), we classified ICC, HCC and non-tumorous tissue with 84.38% accuracy (Table 1). ICC was distinguished from the other 3 sample groups with 78.13% of accuracy (Table 1) using the expression profile of 17 miRNAs (Supplementary Table 4). However mRNA expression combined with the characteristics of the compounds or miRNA expression profile was unable to diagnose ICC with high accuracy (date not shown).

## Discussion

In this study, we performed an integrated analysis of transcriptome and metabolome to clarify the mechanism of ICC and HCC carcinogenesis and to discover novel diagnostic markers for ICC. Using five PCs comprising of 14 compounds, 62 mRNAs and 17 miRNAs, we observed two distinct patterns: tumor/non-tumor, and ICC/non-ICC.

It was difficult to uncover the carcinogenetic mechanism for ICC although each gene expression and the level of each compound could be determined. We used Integrated Molecular Pathway Level Analysis (IMPaLA) to analyze compounds and mRNA and the DIANA-miRPath for miRNA analysis. Aberrant expression of mRNA in ICC was related to aberrant tRNA metabolism, amino acid metabolism, and lipoprotein metabolism (supplementary Fig. 1). Elevated compound levels coincided with lipoprotein and tRNA metabolism (Supplementary Fig. 2). Finally a connection was found between ICC and several miRNA related pathways namely: RNA transport, ribosome biogenesis in eukaryotes, protein export, RNA polymerase, and Aminoacyl-tRNA biosynthesis. Although we did not find that lipoprotein to be a miRNA related pathway, it had a strong connection to miRNA. However lipid metabolism, amino acid metabolism, and RNA metabolism are associated with ICC formation.

In our analysis, there were two primary pathways implicated in ICC. The first is lipid and glucose related pathway. A large number of metabolomic changes were observed in HCC that were relative to cirrhosis or to control subjects (review in^29^). Lipids, bile juice secretion, and ICC were found to be closely inter-related. Taurocholate and phosphatidylcholine have no effect on apolipoprotein B (apo B) secretion but has been known to significantly increase the basolateral secretion of APOA1^30^. Elevated taurine level in ICC in this study coincides with previous reports^14^,^15^. Signs of metabolic remodeling has been detected by metabolomics in the livers of HCC patients, namely a decrease in glucose, citrate, and glycerol 3-phosphate coupled with an increase in pyruvate (signs of the Warburg effect^31^), and a switch from mitochondrial respiration to cytosolic aerobic glycolysis^32^,^33^. In our study, down regulated amino acid metabolism and up regulated G-3-P metabolism were observed in both HCC and ICC. Thus, metabolic reprogramming in HCC and ICC appeared to exhibit a modest Warburg shift towards glycolytic metabolism and a major upregulation of fatty acid catabolism in some tumor types.

The second pathway implicated in ICC is the tRNA related pathway. Aminoacyl-tRNA synthetases (ARSs) are essential and ubiquitous ‘house-keeping’ enzymes responsible for charging amino acids to their cognate tRNAs with a high fidelity and providing the substrates for global protein synthesis^34^. Defects in either canonical or noncanonical ARS functions can cause or contribute to human diseases. ARSs suspected involvement in several types of cancer through aberrant expression and interactions have been previously reported^35^,^36^. Our analysis indicated that in addition to ARS, many RNA regulated pathways were involved in ICC. Many therapeutic reagents that are highly potent also produce adverse side effects such as non steroidal anti-inflammatory drugs associated with increased risk of coronary heart disease, and anti-VEGF-A inhibitors implicated in the disruption of blood vessel maintenance. Novel cancer drug related to ARS might provide a new set of physiologic extracellular or intracellular pathways as the basis for developing novel therapeutics with minimal side effects. The advantage of applying natural secretory or endogenous ARSs (e.g., GARS and EPRS) to clinical use is that they catalyze the ligation of amino acids to their cognate tRNAs with a high fidelity. For example, RS and ARS-interacting multifunctional proteins participate in the formation of Glioblastoma multiforme, therefore these compounds are possible candidates to be used in the development of innovative drugs^36^. Another candidate is human LARS which has the ability to correct mitochondrial dysfunctions caused by tRNA^Leu^UUR^A3233G^ mutation-related neurodegenerative disorder in MELAS syndrome^37^.

Several reports on the origin of hepatocyte and cholangiocyte have led to the understanding that hepatoblasts are bipotent precursors that develop into either hepatocyte (the main epithelial cells in the liver) or cholangiocyte (the epithelial cells lining the intrahepatic biliary ducts). The formation of hepatocyte and cholangiocyte is temporally and spatially separated, which suggests that localized inducers or repressing mechanisms operate to direct the fate of both^38^. Hepatocytes can change into biliary lineage cells when intrahepatic bile duct regeneration is induced, but cholangiocytes cannot proliferate owing to toxic influences^39^. Hepatocytes can transdifferentiate into biliary lineage cells regardless of their position in the hepatic lobule. The location of lineage-converting hepatocytes is likely decided by the nature of the toxins used. Notch-mediated conversion of hepatocytes into biliary lineage cells accelerates ICC formation^24^. Moreover non-B non-C HCC patients were chosen for this study as there would be are no influenced by gene expression or the amount of compounds in hepatic virus B or C infection. In this study, embryological similarity between cholangiocyte and hepatocyte coincided with the similarity between the carcinogenic mechanism of ICC and HCC. Moreover, the difference in clinical malignancy grade between ICC and HCC also coincided with the genetic differences between ICC and HCC.

In this study we were able to diagnose ICC with high accuracy using molecular information from the tumor tissue. Specifically, the serum values of AFP and DCP, and CEA and CA19-9 were used to distinguish HCC and ICC, respectively. However, the specificity and sensitivity for diagnosing HCC by AFP and DCP, and diagnosing ICC by CEA and CA19-9 were not satisfactory. Pathological diagnosis of ICC was complicated because poorly differentiated HCC and ICC had similar pathological findings; as well, there was a type of HCC which had the characteristics of both ICC and HCC. ICC could be diagnosed using miRNA or compounds with an approximate 80% accuracy. To our knowledge this is the first report that has identified a highly specific tumor marker for ICC. Although our sample was not as large as would have been ideal, integrating transcriptome and metabolome analysis creates more reliable results than performing a single analysis such as transriptome or metabolome. We propose that in the future a similar study be done with a larger sample.

In conclusion, we found that there were several common pathways involved in ICC and HCC formation and the clinical and genetic malignant potential of ICC was higher than HCC. Using PCA we also revealed that ICC could be distinguished from non-ICC using the biomarkers we identified. These mRNA, miRNAs, and compounds are a promising start to uncovering novel biomarkers that may lead to therapeutic applications in the future.

## Methods

### Sample preparation

Ten ICC and six HCC samples were obtained by surgical resection (Supplementary Table 1). We created four groups using the ICC/HCC samples and their respective surrounding non-tumor tissues. ICC and HCC were diagnosed using tumor markers (AFP, CEA, CA19-9 and DCP), CT and pathological examination. All samples were negative for HBs-Ag and anti-HCV. All patients provided written informed consent, and the Faculty of Medicine Ethics Committee of Osaka City University approved all aspects of this study in accordance with the Helsinki Declaration.

### RNA preparation and miRNA

Total RNA from tissue samples was prepared using a mirVana miRNA extraction Kit (Ambion, Austin, TX, USA) according to the manufacturer’s instruction. To detect miRNA, 100 ng of RNA was labeled and hybridized using the Human microRNA Microarray Kit (Rel. 12.0) (Agilent Technologies, CA, USA) according to the manufacturer’s protocol for use with Agilent microRNA microarrays Version 1.0. Hybridization signals were detected with Agilent DNA microarray scanner G2505B and the scanned images were analyzed using Agilent feature extraction software (v10.10.1.1). All data were deposited in NCBI’s Gene Expression Omnibus and are accessible through GEO Series accession number GSE57555.

### mRNA microarray

To detect mRNA, 100 ng of RNA was labeled and hybridized using the SurePrint G3 Human GE Microarray Kit (Ver 2.0) (Agilent Technologies, CA, USA) according to the manufacturer’s protocol for use with Agilent microRNA microarrays Version 2.0. Hybridization signals were detected with Agilent DNA microarray scanner G2539A and the scanned images were analyzed using Agilent feature extraction software (v10.10.1.1). All data were deposited in NCBI’s Gene Expression Omnibus and are accessible through GEO Series accession number GSE57555.

### Measurement of metabolites

After surgical resection, the sample aliquots were frozen in liquid nitrogen and then tissue was made into a fine powder using a pestle and mortar in liquid nitrogen. Approximately 50 mg of frozen powder tissue was plunged into 1,500 μL of 50% acetonitrile/Milli-Q water containing internal standards (H3304-1002, Human Metabolome Technologies, Inc., Tsuruoka, Japan) at 0 °C in order to inactivate the enzymes. The tissue was homogenized thrice at 1,500 rpm for 120 sec using a tissue homogenizer (Microsmash MS100R, Tomy Digital Biology Co., Ltd., Tokyo, Japan) and then the homogenate was centrifuged at 2,300 × *g* and 4 °C for 5 min. Subsequently, 800 μL of upper aqueous layer was centrifugally filtered through a Millipore 5-kDa cutoff filter at 9,100 × *g* and 4 °C for 120 min to remove proteins. The filtrate was centrifugally concentrated and re-suspended in 50 μL of Milli-Q water for CE-MS analysis.

Capillary electrophoresis time-of-flight mass spectrometry (CE-TOFMS) was carried out using an Agilent CE Capillary Electrophoresis System equipped with an Agilent 6210 Time of Flight mass spectrometer, Agilent 1100 isocratic HPLC pump, Agilent G1603A CE-MS adapter kit, and Agilent G1607A CE-ESI-MS sprayer kit (Agilent Technologies, Waldbronn, Germany). The systems were controlled by Agilent G2201AA ChemStation software version B.03.01 for CE (Agilent Technologies, Waldbronn, Germany). The metabolites were analyzed using a fused silica capillary (50 μm *i.d.* × 80 cm total length), with commercial electrophoresis buffer (Solution ID: H3301-1001 for cation analysis and H3302-1021 for anion analysis, Human Metabolome Technologies) as the electrolyte. The sample was injected at a pressure of 50 mbar for 10 sec (approximately 10 nL) in cation analysis and 25 sec (approximately 25 nL) in anion analysis. The spectrometer was scanned from *m/z* 50 to 1,000. Other conditions were as previously described^40^,^41^,^42^.

## Additional Information

**How to cite this article**: Murakami, Y. *et al.* Comprehensive analysis of transcriptome and metabolome analysis in Intrahepatic Cholangiocarcinoma and Hepatocellular Carcinoma. *Sci. Rep.* **5**, 16294; doi: 10.1038/srep16294 (2015).

## Supplementary Material

Supplementary Information

## Figures and Tables

**Table 1:** Classifying CC, HCC and non-tumor.

		Result		
		non-tumor	HCC	ICC
A. Using profiling of compounds				
Prediction	non-tumor	14	0	2
	HCC	0	5	0
	ICC	2	1	8
B. Using miRNA expression pattern				
Prediction	non-tumor	13	1	1
	HCC	2	4	1
	ICC	1	1	8
