# Supplementary material for: Comprehensive analysis of transcriptome and metabolome analysis in Intrahepatic Cholangiocarcinoma and Hepatocellular Carcinoma
Source: Sci Rep. 2015 Nov 5;5:16294. doi: 10.1038/srep16294 (PMC4633735; doi:10.1038/srep16294)
Supplement: Supplementary Information [file srep16294-s1.pdf]

Supplementary information

Comprehensive analysis of transcriptome and metabolome analysis in Intrahepatic Cholangiocarcinoma and Hepatocellular Carcinoma

Yoshiki Murakami[1*], Shoji Kubo[2], Akihiro Tamori[1], Saori Itami[1], Etsushi Kawamura[1], Keiko Iwaisako[3], Kazuo Ikeda[4], Norifumi Kawada[1], Takahiro Ochiya[5] and Taguchi Y-h[6]

[1]Department of Hepatology, Graduate School of Medicine, Osaka City University, 1-4-3 Asahimachi, Abeno-ku, Osaka 545-8585, Japan

[2]Department of Hepato-Biliary-Pancreatic Surgery, Graduate School of Medicine, Osaka City University, 1-4-3, Asahimachi, Abeno-ku, Osaka 545-8585, Japan

[3]Department of Target Therapy Oncology, Kyoto University Graduate School of Medicine, 54 Shogoin Kawahara-cho, Sakyo-ku, Kyoto 606-8507, Japan

[4]Department of Anatomy and Regenerative Biology, Graduate School of Medicine, Osaka City University, 1-4-3, Asahimachi, Abeno-ku, Osaka 545-8585, Japan

[5]Division of Molecular and Cellular Medicine, National Cancer Center Research Institute, 5-1-1 Tsukiji, Chuo-ku, Tokyo 104-0045, Japan

[6]Department of Physics, Chuo University, 1-13-27 Kasuga, Bunkyo-ku, Tokyo 112-0003, Japan

Supplementary tables

Supplementary table 1. Clinical information

Supplementary table 2. List of compounds selected by PCA

Supplementary table 3. List of mRNAs selected by PCA

Supplementary table 4. List of miRNAs selected by PCA

Supplementary table 5. List of cancer related pathways based on compound information.

Supplementary table 6. List of cancer related pathways based on mRNA information.

Supplementary table 7. List of cancer related pathways based on miRNA information.

Supplementary figure

Supplementary figure 1. Heatmap of compound

Supplementary figure 2. Heatmap of mRNA

Supplementary methods

Supplementary table 1. Clinical information

Code No.	Sex	Age	CEA (ng/ml)	CA19-9 (ng/ml)	AFP (ng/ml)	DCP (U/ml)	Histology	Tumor size (mm x mm)
ICC								
1	M	74	9.4	34	6	24	M	35 x 45
2	M	32	1.7	1223	3.1	39	M	125 x 95
3	M	79	6.1	15	3.5	306	M	35 x 25
4	M	57	2.5	891	1.7	ND	M	23 x 15
5	M	63	8.6	8	4.9	16	M	21 x 16
6	F	62	3.1	28	3.7	17	M	20 x 18
7	M	39	1.5	105	4.6	21	W	ND
8	M	31	2.1	799	7.5	25	M	21x19
9	F	50	12.8	43566	2	NI	M	54x48
10	M	34	4	44	5.7	29	W	10x15
all		52.1±17.6	5.2±3.9	4671±13673	4.3±1.82	59.6±99.8		39.9±34.5
HCC								
11	M	74	4.9	9	4010.7	515	P	40 x 25
12	M	72	3.4	3	4.3	23	M	55 x 45
13	M	77	20.8	160	1750	8177	M	45 x 30
14	M	71	2.3	10	3.6	767	M	45 x 40

15	M	69	1.5	2	6.1	39365	M	80 x 60
16	F	76	2	2.6	2.9	48911	M	90 x 70
all		73.2±3.1	5.8±7.4	31.1±63.2	962.9±1629.3	16293±21986		59.2±20.8

Abbreviations NI; no information, ND; not determined the size of tumor, Histology; W; well differentiated, M; moderately differentiated, P; poorly differentiated

Supplementary table 2. List of compounds selected by PCA

Accession No.	Name	P-value
C00042	Succinic acid	4.61E-02
C00047	Lysine	3.87E-01
C00051	Glutathione	2.77E-01
C00064	Glutamine	1.37E-01
C00093	Glycerol 3 phosphate	7.67E-06
C00114	Choline	9.61E-02
C00123	Leucine	4.36E-01
C00148	Proline	7.79E-01
C00245	Taurine	1.58E-03
C00262	Hypoxanthine	1.14E-01
C00303	Glutamine	1.37E-01
C00407	Isoleucine	2.90E-01
C00670	Glycerophosphocholine	2.14E-03
C00739	Lysine	3.87E-01
C00763	Proline	7.79E-01
C00819	Glutamine	1.37E-01
C01570	Leucine	4.36E-01
C06418	Isoleucine	2.90E-01

C16434	Isoleucine	2.90E-01
C16435	Proline	7.79E-01
C16439	Leucine	4.36E-01
C16440	Lysine	3.87E-01
unknown	XC0061	1.31E-01
unknown	unknown	1.18E-01

Abbreviation, Accession No. is based on Pubchem.

Supplementary table 3. List of mRNAs selected by PCA

Accession No.	Gene name	Abbreviation	P-value
NM_000035	aldolase B, fructose-bisphosphate	ALDOB	3.95e-04
NM_000039	apolipoprotein A-I	APOA1	3.80e-04
NM_000040	apolipoprotein C-III	APOC3	8.34e-04
NM_000041	apolipoprotein E	APOE	1.30e-03
NM_000042	apolipoprotein H	APOH	1.71e-04
NM_000064	complement component 3	C3	9.44e-04
NM_000150	fucosyltransferase 6	FUT6	1.79e-01
NM_000301	plasminogen	PLG	1.01e-04
NM_000371	transthyretin	TTR	3.16e-03
NM_000412	histidine-rich glycoprotein	HRG	1.81e-04
NM_000477	albumine	ALB	3.89e-03
NM_000509	fibrinogen gamma chain	FGG	2.41e-04
NM_000607	orosomucoid 1	ORM1	7.24e-05
NM_000608	orosomucoid 2	ORM2	3,22e-05
NM_000976	ribosomal protein L12	RPL12	8.38e-04
NM_000978	ribosomal protein L23	RPL23	1.20e-04
NM_001002236	serpin peptidase inhibitor, clade A	SERPINA1	3.10e-03
NM_001003	ribosomal protein, large, P1	RPLP1	6.19e-05

NM_001004	ribosomal protein, large, P2	RPLP2	1.49e-05
NM_001012	ribosomal protein S8	RPS8	2.57e-03
NM_001025195	carboxylesterase 1	CES1	1.38e-03
NM_001030	ribosomal protein S27	RSP27	8.87e-03
NM_001031	ribosomal protein S28	RPS28	2.24e-04
NM_001033045	G protein-coupled receptor 155	GPR155	9.62e-02
NM_001040125	PQ loop repeat containing 2	PQLC2	2.59e-01
NM_001101	actin beta	ACTB	4.22e-05
NM_001113755	thymidine phosphorylase	TYMP	5.12e-01
NM_001190452	MT-RNR2-like 1	MTRNP2L1	2.54e-01
NM_001190470	MT-RNR2-like 2	MTRNP2L2	1.35e-01
NM_001190487	MT-RNR2-like 6	MTRNR2L6	7.29e-02
NM_001195605,	zinc finger protein 865	ZNF865	1.36e-01
NM_001402	eukaryotic translation elongation factor 1 alpha 1	EEF1A1	4.14e-02
NM_001622	alpha-2-HS-glycoprotein	AHSG	8.46e-05
NM_001633	alpha-1-microglobulin/bikunin precursor	AMBP	6.78e-05
NM_001643	apolipoprotein A-II	APOA2	3.78e-04
NM_001645	apolipoprotein C-I	APOC1	5.72e-04
NM_001733	complement component 1, r subcomponent	C1R	2.54e-05
NM_002116	major histocompatibility complex, class I, A	HLA-A	1.28e-02

NM_002218	inter-alpha-trypsin inhibitor heavy chain family, member 4	ITIH4	2.79e-05
NM_002952	ribosomal protein S2	RPS2	3.27e-05
NM_005143	haptoglobin	HP	3.90e-04
NM_005946	metallothionein 1A	MT1A	1.03e-04
NM_005953	metallothionein 2A	MT2A	9.58e-04
NM_006744	retinol binding protein 4, plasma	RBP4	1.04e-04
NM_014272	ADAM metallopeptidase with thrombospondin type 1 motif, 7	ADAMTS7	8.27e-01
NM_017781	cytochrome P450, family 2, subfamily W, polypeptide 1	CYP2W1	4.30e-01
NM_020682	arsenite methyltransferase	AS3MT	1.97e-01
NM_020995	haptoglobin-related protein	HRP	2.60e-05
NM_021009	ubiquitin C	UBC	1.14e-01
NM_022551	ribosomal protein S18	RPS18	1.36e-01
NM_030885	microtubule-associated protein 4	MAP4	3.69e-01
NM_033251	ribosomal protein L1	RPL13	3.08e-03
NM_172002	HscB mitochondrial iron-sulfur cluster co-chaperone	HSCB	1.08e-01
NM_178352	late cornified envelope 1D	LCE1D	8.73e-01
NM_213606	solute carrier family 16, member 12	SLC16A12	1.21e-01

Abbreviation: This list includes some overlapping microarray probe (9 APOA1, 2 MTRNR2L2, and 2 RPL2

Supplementary table 4. List of miRNAs selected by PCA

miRNA	p-value	miRNA	p-value
hsa-let-7a-5p	0.00E+00	hsa-miR-4454	2.85E-04
hsa-let-7b-5p	0.00E+00	hsa-miR-4459	0.00E+00
hsa-miR-122-5p	0.00E+00	hsa-miR-4516	0.00E+00
hsa-miR-16-5p	6.66E-16	hsa-miR-451a	0.00E+00
hsa-miR-21-5p	0.00E+00	hsa-miR-5100	9.73E-06
hsa-miR-29a-3p	1.84E-14	hsa-miR-6087	1.60E-01
hsa-miR-3917	5.40E-01	hsa-miR-6089	0.00E+00
hsa-miR-3960	0.00E+00	hsa-miR-642a-3p	0.00E+00
hsa-miR-4286	3.24E-12		

Supplementary table 5. List of cancer related pathway based on compound information.

Name	Source	Overlapping metabolites	No. of metabolites	P-value
Amine compound SLC transporters	Reactome	C00114;C00064;C00148;C00245;C00407;C00047;C00123	35 (35)	4.31E-08
ABC transporters - Homo sapiens (human)	KEGG	C00064;C00114;C00148;C00245;C00093;C00407;C00051;C00047;C00123	122 (122)	9.47E-08
Transport of glucose and other sugars_ bile salts and organic acids_ metal ions and amine compounds	Reactome	C00064;C00114;C00148;C00245;C00407;C00042;C00047;C00123	78 (83)	9.47E-08
leukotriene biosynthesis	HumanCyc	C00064;C00148;C00407;C00051;C00047;C00123	29 (30)	2.71E-07
γ-glutamyl cycle	HumanCyc	C00064;C00148;C00407;C00051;C00047;C00123	29 (29)	2.71E-07
Glutathione synthesis and recycling	Reactome	C00064;C00148;C00407;C00051;C00047;C00123	30 (31)	2.82E-07
Na+/Cl- dependent neurotransmitter transporters	Reactome	C00064;C00148;C00245;C00407;C00047;C00123	31 (31)	2.88E-07
Amino acid transport across the plasma membrane	Reactome	C00064;C00148;C00245;C00407;C00047;C00123	32 (32)	2.88E-07
SLC-mediated transmembrane transport	Reactome	C00114;C00064;C00148;C00262;C00245;	158 (164)	2.88E-07

		C00407;C00042;C00047;C00123		
Glutathione conjugation	Reactome	C00064;C00148;C00407;C00051;C00047;C00123	36 (40)	5.48E-07
Transmembrane transport of small molecules	Reactome	C00114;C00064;C00148;C00262;C00245;C00407;C00042;C00047;C00123	184 (195)	9.27E-07
Endosomal/Vacuolar pathway	Reactome	C00064;C00148;C00047;C00123;C00407	20 (20)	1.49E-06
Amino acid and oligopeptide SLC transporters	Reactome	C00064;C00148;C00245;C00407;C00047;C00123	45 (45)	1.67E-06
Proton/oligonucleotide cotransporters	Reactome	C00064;C00148;C00047;C00123;C00407	21 (21)	1.67E-06
Transport of inorganic cations/anions and amino acids/oligopeptides	Reactome	C00064;C00148;C00245;C00407;C00047;C00123	48 (48)	2.25E-06
tRNA charging	HumanCyc	C00064;C00148;C00047;C00123;C00407	24 (24)	3.03E-06
Antigen processing-Cross presentation	Reactome	C00064;C00148;C00047;C00123;C00407	29 (29)	7.89E-06
Transport of inorganic cations-anions and amino acids-oligopeptides	Wikipathways	C00064;C00148;C00407;C00123;C00047	31 (32)	1.06E-05
Metabolism of amino acids and derivatives	Reactome	C00064;C00148;C00245;C00407;C00042;C00051;C00047;C00123	181 (190)	1.32E-05
<i>S</i>-methyl-5-thio-α-D-ribose 1-phosphate degradation	HumanCyc	C00064;C00148;C00047;C00123;C00407	35 (35)	1.72E-05
Class I MHC mediated antigen processing &	Reactome	C00064;C00148;C00047;C00123;C00407	35 (35)	1.72E-05

presentation				
Immune System	Reactome	C00114;C00064;C00148;C00407;C00047;C00123	87 (102)	5.84E-05
Protein digestion and absorption - Homo sapiens (human)	KEGG	C00064;C00148;C00407;C00123;C00047	47 (47)	7.25E-05
Adaptive Immune System	Reactome	C00064;C00148;C00047;C00123;C00407	48 (48)	7.74E-05
Gene Expression	Reactome	C00064;C00148;C00407;C00042;C00047;C00123	94 (100)	8.17E-05
Phase II conjugation	Wikipathways	C00064;C00148;C00407;C00051;C00047;C00123	95 (110)	8.37E-05
Aminoacyl-tRNA biosynthesis - Homo sapiens (human)	KEGG	C00064;C00148;C00407;C00123;C00047	52 (52)	0.000104
Metabolism	Reactome	C00114;C00064;C00148;C00262;C00245;C00093;C00407;C00670;C00042;C00051;C00047;C00123	794 (1000)	0.000107
Phase II conjugation	Reactome	C00064;C00148;C00407;C00051;C00047;C00123	114 (140)	0.000222
Hydrolysis of LPC	Reactome	C00114;C00093;C00670	8 (8)	0.000254
tRNA Aminoacylation	Wikipathways	C00064;C00148;C00407;C00123;C00047	65 (65)	0.000254
Cytosolic tRNA aminoacylation	Reactome	C00064;C00148;C00047;C00123;C00407	65 (65)	0.000254

Mitochondrial tRNA aminoacylation	Reactome	C00064;C00148;C00047;C00123;C00407	65 (65)	0.000254
tRNA Aminoacylation	Reactome	C00064;C00148;C00047;C00123;C00407	65 (65)	0.000254
Mineral absorption - Homo sapiens (human)	KEGG	C00064;C00148;C00407;C00123	29 (29)	0.000276
Metabolism of amino acids and derivatives	Wikipathways	C00064;C00148;C00245;C00042;C00051;C00047	173 (186)	0.00203
One carbon donor	Wikipathways	C00114;C00051;C00245	19 (23)	0.00275
Doxycycline Action Pathway	SMPDB	C00148;C00407;C00123	20 (20)	0.00275
Demeclocycline Action Pathway	SMPDB	C00148;C00407;C00123	20 (20)	0.00275
Oxytetracycline Action Pathway	SMPDB	C00148;C00407;C00123	20 (20)	0.00275
Minocycline Action Pathway	SMPDB	C00148;C00407;C00123	20 (20)	0.00275
Lymecycline Action Pathway	SMPDB	C00148;C00407;C00123	20 (20)	0.00275
Tetracycline Action Pathway	SMPDB	C00148;C00407;C00123	20 (20)	0.00275
Clomocycline Action Pathway	SMPDB	C00148;C00407;C00123	20 (20)	0.00275
Clarithromycin Action Pathway	SMPDB	C00148;C00407;C00123	20 (20)	0.00275
Clindamycin Action Pathway	SMPDB	C00148;C00407;C00123	20 (20)	0.00275
Azithromycin Action Pathway	SMPDB	C00148;C00407;C00123	20 (20)	0.00275
Streptomycin Action Pathway	SMPDB	C00148;C00407;C00123	20 (20)	0.00275
Spectinomycin Action Pathway	SMPDB	C00148;C00407;C00123	20 (20)	0.00275
Kanamycin Action Pathway	SMPDB	C00148;C00407;C00123	20 (20)	0.00275
Gentamicin Action Pathway	SMPDB	C00148;C00407;C00123	20 (20)	0.00275

Netilmicin Action Pathway	SMPDB	C00148;C00407;C00123	20 (20)	0.00275
Neomycin Action Pathway	SMPDB	C00148;C00407;C00123	20 (20)	0.00275
Roxithromycin Action Pathway	SMPDB	C00148;C00407;C00123	20 (20)	0.00275
Erythromycin Action Pathway	SMPDB	C00148;C00407;C00123	20 (20)	0.00275
Amikacin Action Pathway	SMPDB	C00148;C00407;C00123	20 (20)	0.00275
Telithromycin Action Pathway	SMPDB	C00148;C00407;C00123	20 (20)	0.00275
Biological oxidations	Reactome	C00064;C00148;C00407;C00051;C00047;C00123	202 (252)	0.00306
Glucose Homeostasis	Wikipathways	C00262;C00407;C00047	21 (21)	0.0031
Urea cycle and metabolism of arginine_ proline_ glutamate_ aspartate and asparagine	EHMN	C00042;C00064;C00148;C00051;C00047	125 (125)	0.0036
Amino acid conjugation	Wikipathways	C00064;C00245	5 (5)	0.00715
Transport of glucose and other sugars_ bile salts and organic acids_ metal ions and amine compounds	Wikipathways	C00114;C00042;C00148	29 (29)	0.00795
Neurotransmitter Release Cycle	Reactome	C00114;C00042;C00064	30 (30)	0.00867
Glycine_ serine_ alanine and threonine metabolism	EHMN	C00114;C00064;C00051;C00047	88 (88)	0.013
Glycerophospholipid metabolism	EHMN	C00114;C00064;C00093;C00670	96 (96)	0.0179
GABAergic synapse - Homo sapiens (human)	KEGG	C00064;C00042	9 (9)	0.0232

Acetylcholine Synthesis	Wikipathways	C00114;C00670	9 (9)	0.0232
Homocarnosinosis	SMPDB	C00042;C00064;C00051	48 (48)	0.0306
Hyperinsulinism-Hyperammonemia Syndrome	SMPDB	C00042;C00064;C00051	48 (48)	0.0306
Succinic semialdehyde dehydrogenase deficiency	SMPDB	C00042;C00064;C00051	48 (48)	0.0306
4-Hydroxybutyric Aciduria/Succinic Semialdehyde Dehydrogenase Deficiency	SMPDB	C00042;C00064;C00051	48 (48)	0.0306
Glutamate Metabolism	SMPDB	C00042;C00064;C00051	48 (48)	0.0306
2-Hydroxyglutric Aciduria (D And L Form)	SMPDB	C00042;C00064;C00051	48 (48)	0.0306
Transmission across Chemical Synapses	Reactome	C00064;C00114;C00042	51 (51)	0.0318
Neuronal System	Reactome	C00064;C00114;C00042	51 (51)	0.0318
Prolinemia Type II	SMPDB	C00042;C00148;C00763	52 (52)	0.0318
Prolidase Deficiency (PD)	SMPDB	C00042;C00148;C00763	52 (52)	0.0318
Arginine and Proline Metabolism	SMPDB	C00042;C00148;C00763	52 (52)	0.0318
Hyperprolinemia Type I	SMPDB	C00042;C00148;C00763	52 (52)	0.0318
Hyperprolinemia Type II	SMPDB	C00042;C00148;C00763	52 (52)	0.0318
Ornithine Aminotransferase Deficiency (OAT Deficiency)	SMPDB	C00042;C00148;C00763	52 (52)	0.0318
Arginine: Glycine Amidinotransferase Deficiency (AGAT Deficiency)	SMPDB	C00042;C00148;C00763	52 (52)	0.0318

Glycerophospholipid metabolism - Homo sapiens (human)	KEGG	C00114;C00093;C00670	52 (52)	0.0318
Hyperornithinemia with gyrate atrophy (HOGA)	SMPDB	C00042;C00148;C00763	52 (52)	0.0318
Creatine deficiency_ guanidinoacetate methyltransferase deficiency	SMPDB	C00042;C00148;C00763	52 (52)	0.0318
L-arginine:glycine amidinotransferase deficiency	SMPDB	C00042;C00148;C00763	52 (52)	0.0318
Hyperornithinemia-hyperammonemia-homocitrullinuria [HHH-syndrome]	SMPDB	C00042;C00148;C00763	52 (52)	0.0318
Guanidinoacetate Methyltransferase Deficiency (GAMT Deficiency)	SMPDB	C00042;C00148;C00763	52 (52)	0.0318
D-Glutamine and D-glutamate metabolism - Homo sapiens (human)	KEGG	C00064;C00819	12 (12)	0.0318
Synthesis of PG	Reactome	C00114;C00093	13 (13)	0.0354
Glycerophospholipid biosynthesis	Wikipathways	C00114;C00093;C00670	56 (64)	0.0354
Trans-sulfuration pathway	Wikipathways	C00051;C00245	14 (14)	0.0354
Isovaleric Aciduria	SMPDB	C00042;C00407;C00123	58 (58)	0.0354
3-Methylcrotonyl Coa Carboxylase Deficiency Type I	SMPDB	C00042;C00407;C00123	58 (58)	0.0354
Propionic Acidemia	SMPDB	C00042;C00407;C00123	58 (58)	0.0354

Maple Syrup Urine Disease	SMPDB	C00042;C00407;C00123	58 (58)	0.0354
3-Hydroxy-3-Methylglutaryl-CoA Lyase Deficiency	SMPDB	C00042;C00407;C00123	58 (58)	0.0354
Isobutyryl-coa dehydrogenase deficiency	SMPDB	C00042;C00407;C00123	58 (58)	0.0354
3-hydroxyisobutyric aciduria	SMPDB	C00042;C00407;C00123	58 (58)	0.0354
3-hydroxyisobutyric acid dehydrogenase deficiency	SMPDB	C00042;C00407;C00123	58 (58)	0.0354
Isovaleric acidemia	SMPDB	C00042;C00407;C00123	58 (58)	0.0354
Methylmalonate Semialdehyde Dehydrogenase Deficiency	SMPDB	C00042;C00407;C00123	58 (58)	0.0354
Methylmalonic Aciduria	SMPDB	C00042;C00407;C00123	58 (58)	0.0354
3-Methylglutaconic Aciduria Type IV	SMPDB	C00042;C00407;C00123	58 (58)	0.0354
3-Methylglutaconic Aciduria Type III	SMPDB	C00042;C00407;C00123	58 (58)	0.0354
Beta-Ketothiolase Deficiency	SMPDB	C00042;C00407;C00123	58 (58)	0.0354
Glycerophospholipid biosynthesis	Reactome	C00114;C00093;C00670	58 (63)	0.0354
3-Methylglutaconic Aciduria Type I	SMPDB	C00042;C00407;C00123	58 (58)	0.0354
Valine_ Leucine and Isoleucine Degradation	SMPDB	C00042;C00407;C00123	58 (58)	0.0354
2-Methyl-3-Hydroxybutryl CoA Dehydrogenase Deficiency	SMPDB	C00042;C00407;C00123	58 (58)	0.0354
Metabolism of lipids and lipoproteins	Reactome	C00114;C00245;C00093;C00670;C00042;	358 (443)	0.0372

		C00051		
Synthesis of PA	Reactome	C00114;C00093	15 (15)	0.04
Phospholipid metabolism	Reactome	C00114;C00093;C00670	66 (71)	0.0498
Purine metabolism	Reactome	C00064;C00051;C00262	66 (67)	0.0498

Supplementary table 6. List of cancer related pathway based on mRNA information.

Name	Source	Overlapping genes	No. of genes	p-value
Peptide chain elongation	Reactome	NM_001003;NM_000978;NM_002952;NM_022551;NM_001030;NM_000976;NM_033251;NM_001012;NM_001402;NM_001031;NM_001004	95 (95)	5.64E-10
Eukaryotic Translation Elongation	Reactome	NM_001003;NM_000978;NM_002952;NM_022551;NM_001030;NM_000976;NM_033251;NM_001012;NM_001402;NM_001031;NM_001004	100 (100)	5.64E-10
Cytoplasmic Ribosomal Proteins	Wikipathways	NM_001003;NM_000978;NM_002952;NM_022551;NM_001030;NM_000976;NM_033251;NM_001012;NM_001031;NM_001004	88 (88)	3.62E-09
Eukaryotic Translation Termination	Reactome	NM_001003;NM_000978;NM_002952;NM_022551;NM_001030;NM_000976;NM_033251;NM_001012;NM_001031;NM_001004	94 (94)	5.34E-09
Nonsense Mediated Decay (NMD) independent of the Exon Junction Complex (EJC)	Reactome	NM_001003;NM_000978;NM_002952;NM_022551;NM_001030;NM_000976;NM_033251;NM_001012;NM_001031;NM_001004	99 (99)	7.25E-09
Formation of a pool of free 40S subunits	Reactome	NM_001003;NM_000978;NM_002952;NM_022551;NM_001030;NM_000976;NM_033251;NM_001012;NM_001031;NM_001004	106 (106)	1.21E-08
Nonsense Mediated Decay (NMD) enhanced	Reactome	NM_001003;NM_000978;NM_002952;NM_0225	110 (110)	1.32E-08

by the Exon Junction Complex (EJC)		51;NM_001030;NM_000976;NM_033251;NM_001012;NM_001031;NM_001004		
Nonsense-Mediated Decay (NMD)	Reactome	NM_001003;NM_000978;NM_002952;NM_022551;NM_001030;NM_000976;NM_033251;NM_001012;NM_001031;NM_001004	110 (110)	1.32E-08
3_ -UTR-mediated translational regulation	Reactome	NM_001003;NM_000978;NM_002952;NM_022551;NM_001030;NM_000976;NM_033251;NM_001012;NM_001031;NM_001004	116 (116)	1.64E-08
L13a-mediated translational silencing of Ceruloplasmin expression	Reactome	NM_001003;NM_000978;NM_002952;NM_022551;NM_001030;NM_000976;NM_033251;NM_001012;NM_001031;NM_001004	116 (116)	1.64E-08
GTP hydrolysis and joining of the 60S ribosomal subunit	Reactome	NM_001003;NM_000978;NM_002952;NM_022551;NM_001030;NM_000976;NM_033251;NM_001012;NM_001031;NM_001004	117 (117)	1.64E-08
Translation	Reactome	NM_001003;NM_000978;NM_002952;NM_022551;NM_001030;NM_000976;NM_033251;NM_001012;NM_001402;NM_001031;NM_001004	160 (160)	1.64E-08
SRP-dependent cotranslational protein targeting to membrane	Reactome	NM_001003;NM_000978;NM_002952;NM_022551;NM_001030;NM_000976;NM_033251;NM_001012;NM_001031;NM_001004	118 (118)	1.64E-08
Cap-dependent Translation Initiation	Reactome	NM_001003;NM_000978;NM_002952;NM_0225	124 (124)	2.34E-08

		51;NM_001030;NM_000976;NM_033251;NM_001012;NM_001031;NM_001004		
Eukaryotic Translation Initiation	Reactome	NM_001003;NM_000978;NM_002952;NM_022551;NM_001030;NM_000976;NM_033251;NM_001012;NM_001031;NM_001004	124 (124)	2.34E-08
Ribosome - Homo sapiens (human)	KEGG	NM_001003;NM_001004;NM_002952;NM_022551;NM_000976;NM_001030;NM_033251;NM_001012;NM_001031;NM_000978	133 (134)	4.42E-08
Scavenging of heme from plasma	Reactome	NM_005143;NM_020995;NM_001633;NM_000477;NM_000039	12 (12)	2.06E-07
Metabolism of mRNA	Reactome	NM_001003;NM_001004;NM_002952;NM_022551;NM_001030;NM_000976;NM_033251;NM_001012;NM_001031;NM_000978	178 (178)	6.87E-07
Metabolism of proteins	Reactome	NM_001003;NM_172002;NM_001004;NM_000301;NM_000978;NM_002952;NM_022551;NM_000976;NM_001030;NM_033251;NM_001031;NM_001402;NM_001101;NM_001012;NM_014272	598 (598)	3.06E-06
Binding and Uptake of Ligands by Scavenger Receptors	Reactome	NM_001633;NM_005143;NM_000039;NM_020995;NM_000477;NM_000041	41 (41)	3.52E-06
Retinoid metabolism and transport	Reactome	NM_000371;NM_000039;NM_001643;NM_0000	42 (42)	3.90E-06

		40;NM_000041;NM_006744		
Metabolism of RNA	Reactome	NM_001003;NM_001004;NM_002952;NM_022551;NM_001030;NM_000976;NM_033251;NM_001012;NM_001031;NM_000978	226 (226)	5.57E-06
Lipoprotein metabolism	Reactome	NM_001643;NM_000040;NM_000041;NM_000477;NM_000039	30 (30)	2.59E-05
Statin Pathway	Wikipathways	NM_001643;NM_000040;NM_000041;NM_001645;NM_000039	31 (31)	2.95E-05
HDL-mediated lipid transport	Reactome	NM_000040;NM_000041;NM_000477;NM_000039	15 (15)	6.02E-05
Chylomicron-mediated lipid transport	Reactome	NM_001643;NM_000040;NM_000041;NM_000039	17 (17)	0.0001
Platelet degranulation	Reactome	NM_000412;NM_000039;NM_001002236;NM_000477;NM_000509;NM_000301	82 (82)	0.000178
Response to elevated platelet cytosolic Ca2+	Reactome	NM_000412;NM_000039;NM_001002236;NM_000477;NM_000509;NM_000301	87 (87)	0.000243
Lipid digestion_ mobilization_ and transport	Reactome	NM_001643;NM_000040;NM_000041;NM_000477;NM_000039	50 (50)	0.000286
Diseases associated with visual transduction	Reactome	NM_000371;NM_000039;NM_001643;NM_000040;NM_000041;NM_006744	96 (96)	0.000382

Visual phototransduction	Reactome	NM_000371;NM_000039;NM_001643;NM_000040;NM_000041;NM_006744	96 (96)	0.000382
Complement and Coagulation Cascades	Wikipathways	NM_001643;NM_001002236;NM_001733;NM_000301;NM_000064	54 (54)	0.000382
Formation of the ternary complex_ and subsequently_ the 43S complex	Reactome	NM_001030;NM_001031;NM_002952;NM_001012;NM_022551	55 (55)	0.000397
Statin Pathway_ Pharmacodynamics	PharmGKB	NM_000040;NM_000041;NM_001645;NM_000039	25 (25)	0.000397
Translation initiation complex formation	Reactome	NM_001030;NM_001031;NM_002952;NM_001012;NM_022551	62 (62)	0.000677
Ribosomal scanning and start codon recognition	Reactome	NM_001030;NM_001031;NM_002952;NM_001012;NM_022551	62 (62)	0.000677
Activation of the mRNA upon binding of the cap-binding complex and eIFs_ and subsequent binding to 43S	Reactome	NM_001030;NM_001031;NM_002952;NM_001012;NM_022551	63 (63)	0.000713
Complement and coagulation cascades - Homo sapiens (human)	KEGG	NM_001002236;NM_000509;NM_001733;NM_000301;NM_000064	69 (69)	0.00109
FOXA2 and FOXA3 transcription factor networks	PID	NM_000477;NM_000371;NM_000035;NM_000039	45 (45)	0.00383
Complement	Wikipathways	NM_000509;NM_000301;NM_000477;NM_0000	95 (95)	0.0049

		64;NM_000039		
Vitamin B12 Metabolism	Wikipathways	NM_000301;NM_000041;NM_000477;NM_000039	51 (51)	0.00599
Staphylococcus aureus infection - Homo sapiens (human)	KEGG	NM_000509;NM_001733;NM_000301;NM_000064	57 (57)	0.00907
Capecitabine Metabolism Pathway	SMPDB	NM_001113755;NM_001025195	5 (5)	0.0152
Capecitabine Action Pathway	SMPDB	NM_001113755;NM_001025195	5 (5)	0.0152
PPAR signaling pathway - Homo sapiens (human)	KEGG	NM_001643;NM_000040;NM_021009;NM_000039	69 (69)	0.0178
Platelet activation_ signaling and aggregation	Reactome	NM_000412;NM_000039;NM_001002236;NM_000477;NM_000509;NM_000301	208 (208)	0.0207
Gene Expression	Reactome	NM_001003;NM_001004;NM_002952;NR_003287;NM_022551;NM_000976;NM_021009;NM_001030;NM_033251;NM_001031;NM_001402;NM_001012;NM_000978	1103 (1104)	0.0471

Supplementary table 7. The pathway related to miRNAs

KEGG pathway	p-value	#genes	#miRNAs
p53 signaling pathway	2.54E-25	32	7
RNA transport	8.37E-23	52	6
Hepatitis B	1.10E-19	49	7
Prion diseases	1.13E-19	11	5
Ribosome biogenesis in eukaryotes	1.25E-17	30	5
Prostate cancer	1.27E-15	32	7
Bladder cancer	1.15E-14	18	6
Colorectal cancer	1.78E-13	23	7
Small cell lung cancer	1.22E-12	28	7
Cell cycle	1.41E-10	35	7
Pancreatic cancer	2.29E-10	25	7
Pathways in cancer	3.30E-10	74	7
Chronic myeloid leukemia	3.30E-10	24	7
Protein export	9.00E-10	11	4
Ribosome	1.99E-09	27	5
Insulin signaling pathway	7.04E-09	36	7
Legionellosis	8.10E-09	19	5
PI3K-Akt signaling pathway	1.92E-08	72	7

Glioma	1.94E-08	23	7
Non-small cell lung cancer	2.97E-08	18	7
Melanoma	3.49E-07	20	7
Epstein-Barr virus infection	5.74E-07	47	7
Protein processing in endoplasmic reticulum	1.99E-06	40	7
Ubiquitin mediated proteolysis	3.21E-06	34	7
Endometrial cancer	5.73E-06	16	7
Viral carcinogenesis	5.31E-05	47	7
Acute myeloid leukemia	5.51E-05	16	6
Chagas disease (American trypanosomiasis)	5.51E-05	25	7
RNA polymerase	1.05e-04	10	4
HIF-1 signaling pathway	2.00e-04	26	7
Influenza A	2.00e-04	37	7
Focal adhesion	3.08e-04	41	7
Herpes simplex infection	4.47e-04	40	7
Lysine degradation	5.56e-04	14	5
Toxoplasmosis	1.28e-03	27	7
Hepatitis C	1.96e-03	27	7
Progesterone-mediated oocyte maturation	2.00e-03	19	7
Aminoacyl-tRNA biosynthesis	2.82e-03	15	4

mTOR signaling pathway	2.82e-03	15	8
Amyotrophic lateral sclerosis (ALS)	2.93e-03	14	7
Citrate cycle (TCA cycle)	4.16e-03	9	4
ErbB signaling pathway	4.46e-03	18	7
Oocyte meiosis	4.47e-03	23	7
Salmonella infection	4.84e-03	18	7
Viral myocarditis	4.88e-03	16	7
TGF-beta signaling pathway	5.18e-03	20	6
Thyroid cancer	6.04e-03	8	4
Measles	6.07e-03	29	7
Neurotrophin signaling pathway	6.31e-03	25	7
HTLV-I infection	6.61e-03	47	7
Basal transcription factors	7.16e-03	11	5
Shigellosis	7.16e-03	15	7
B cell receptor signaling pathway	7.81e-03	17	6
Spliceosome	7.94e-03	28	5
Epithelial cell signaling in Helicobacter pylori infection	1.13e-02	15	7
Adipocytokine signaling pathway	1.51e-02	15	6
GnRH signaling pathway	2.38e-02	18	7
Mineral absorption	2.60e-02	12	6

Butanoate metabolism	2.64e-02	7	5
Pyruvate metabolism	3.96e-02	10	5
Hypertrophic cardiomyopathy (HCM)	4.15e-02	16	7
Transcriptional misregulation in cancer	4.63e-02	34	7

Abbreviations, #genes: number of genes related to this pathway, #miRNA: number of miRNA related to this pathway

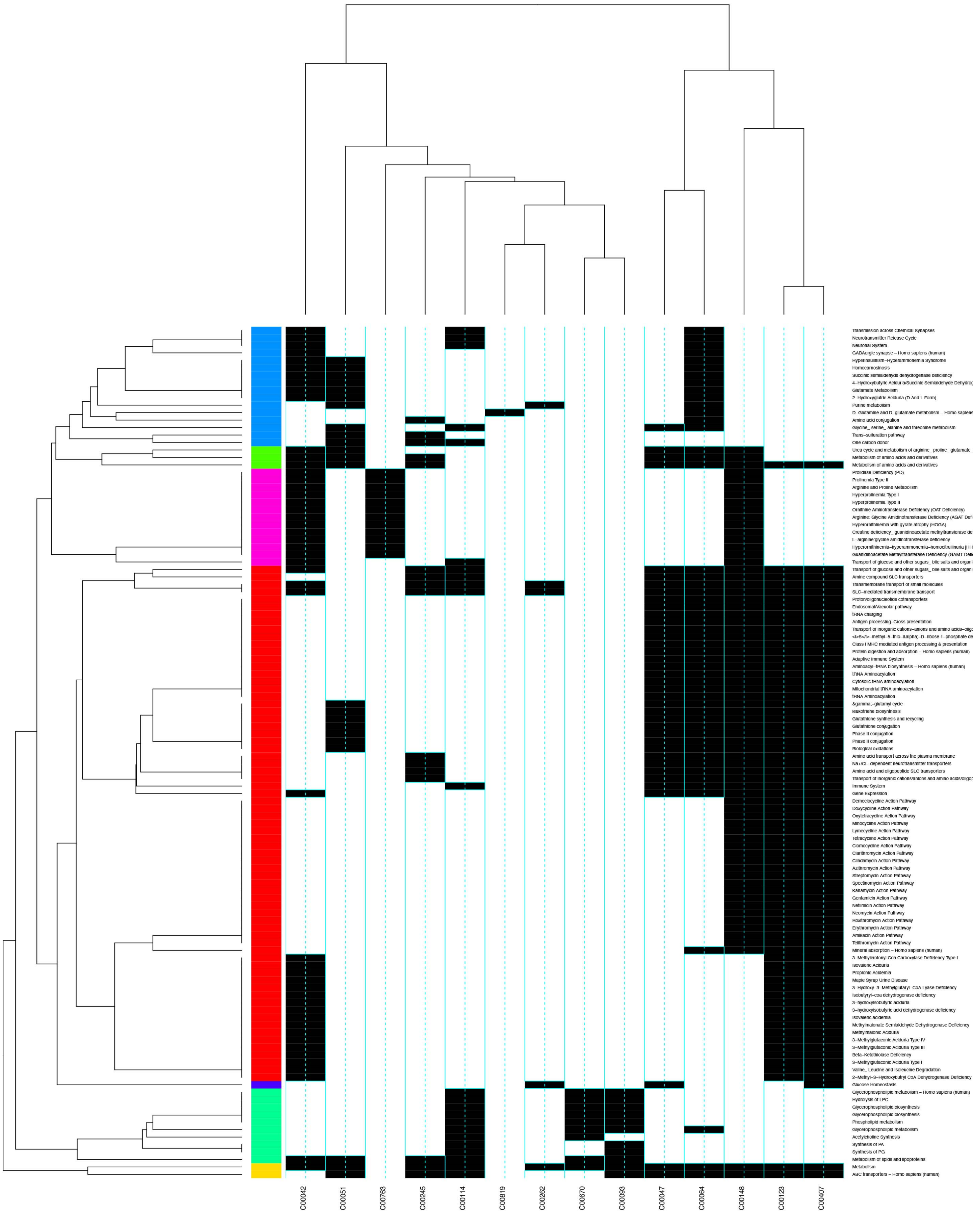

Transmission across Chemical Synapses
Neurotransmitter Release Cycle
Neuronal System
GABAergic synapse – Homo sapiens (human)
Hyperinsulinism–Hyperammonemia Syndrome
Homocarnosinosis
Succinic semialdehyde dehydrogenase deficiency
4–Hydroxybutyric Aciduria/Succinic Semialdehyde Dehydrog
Glutamate Metabolism
2–Hydroxyglutric Aciduria (D And L Form)
Purine metabolism
D–Glutamine and D–glutamate metabolism – Homo sapiens
Amino acid conjugation
Glycine_ serine_ alanine and threonine metabolism
Trans–sulfuration pathway
One carbon donor
Urea cycle and metabolism of arginine_ proline_ glutamate_
Metabolism of amino acids and derivatives
Metabolism of amino acids and derivatives
Prolidase Deficiency (PD)
Prolinemia Type II
Arginine and Proline Metabolism
Hyperprolinemia Type I
Hyperprolinemia Type II
Ornithine Aminotransferase Deficiency (OAT Deficiency)
Arginine: Glycine Amidinotransferase Deficiency (AGAT Defi
Hyperornithinemia with gyrate atrophy (HOGA)
Creatine deficiency_ guanidinoacetate methyltransferase de
L–arginine:glycine amidinotransferase deficiency
Hyperornithinemia–hyperammonemia–homocitrullinuria [HH
Guanidinoacetate Methyltransferase Deficiency (GAMT Defi
Transport of glucose and other sugars_ bile salts and organi
Transport of glucose and other sugars_ bile salts and organi
Amine compound SLC transporters
Transmembrane transport of small molecules
SLC–mediated transmembrane transport
Proton/oligonucleotide cotransporters
Endosomal/Vacuolar pathway
tRNA charging
Antigen processing–Cross presentation
Transport of inorganic cations–anions and amino acids–oligo
<i>S</i>–methyl–5–thio–α–D–ribose 1–phosphate de
Class I MHC mediated antigen processing & presentation
Protein digestion and absorption – Homo sapiens (human)
Adaptive Immune System
Aminoacyl–tRNA biosynthesis – Homo sapiens (human)
tRNA Aminoacylation
Cytosolic tRNA aminoacylation
Mitochondrial tRNA aminoacylation
tRNA Aminoacylation
γ–glutamyl cycle
leukotriene biosynthesis
Glutathione synthesis and recycling
Glutathione conjugation
Phase II conjugation
Phase II conjugation
Biological oxidations
Amino acid transport across the plasma membrane
Na+/Cl– dependent neurotransmitter transporters
Amino acid and oligopeptide SLC transporters
Transport of inorganic cations/anions and amino acids/oligop
Immune System
Gene Expression
Demeclocycline Action Pathway
Doxycycline Action Pathway
Oxytetracycline Action Pathway
Minocycline Action Pathway
Lymecycline Action Pathway
Tetracycline Action Pathway
Clomocycline Action Pathway
Clarithromycin Action Pathway
Clindamycin Action Pathway
Azithromycin Action Pathway
Streptomycin Action Pathway
Spectinomycin Action Pathway
Kanamycin Action Pathway
Gentamicin Action Pathway
Netilmicin Action Pathway
Neomycin Action Pathway
Roxithromycin Action Pathway
Erythromycin Action Pathway
Amikacin Action Pathway
Telithromycin Action Pathway
Mineral absorption – Homo sapiens (human)
3–Methylcrotonyl Coa Carboxylase Deficiency Type I
Isovaleric Aciduria
Propionic Acidemia
Maple Syrup Urine Disease
3–Hydroxy–3–Methylglutaryl–CoA Lyase Deficiency
Isobutyryl–coa dehydrogenase deficiency
3–hydroxyisobutyric aciduria
3–hydroxyisobutyric acid dehydrogenase deficiency
Isovaleric acidemia
Methylmalonate Semialdehyde Dehydrogenase Deficiency
Methylmalonic Aciduria
3–Methylglutaconic Aciduria Type IV
3–Methylglutaconic Aciduria Type III
Beta–Ketothiolase Deficiency
3–Methylglutaconic Aciduria Type I
Valine_ Leucine and Isoleucine Degradation
2–Methyl–3–Hydroxybutryl CoA Dehydrogenase Deficiency
Glucose Homeostasis
Glycerophospholipid metabolism – Homo sapiens (human)
Hydrolysis of LPC
Glycerophospholipid biosynthesis
Glycerophospholipid biosynthesis
Phospholipid metabolism
Glycerophospholipid metabolism
Acetylcholine Synthesis
Synthesis of PA
Synthesis of PG
Metabolism of lipids and lipoproteins
Metabolism
ABC transporters – Homo sapiens (human)
C00042
C00051
C00763
C00245
C00114
C00819
C00262
C00670
C00093
C00047
C00064
C00148
C00123
C00407

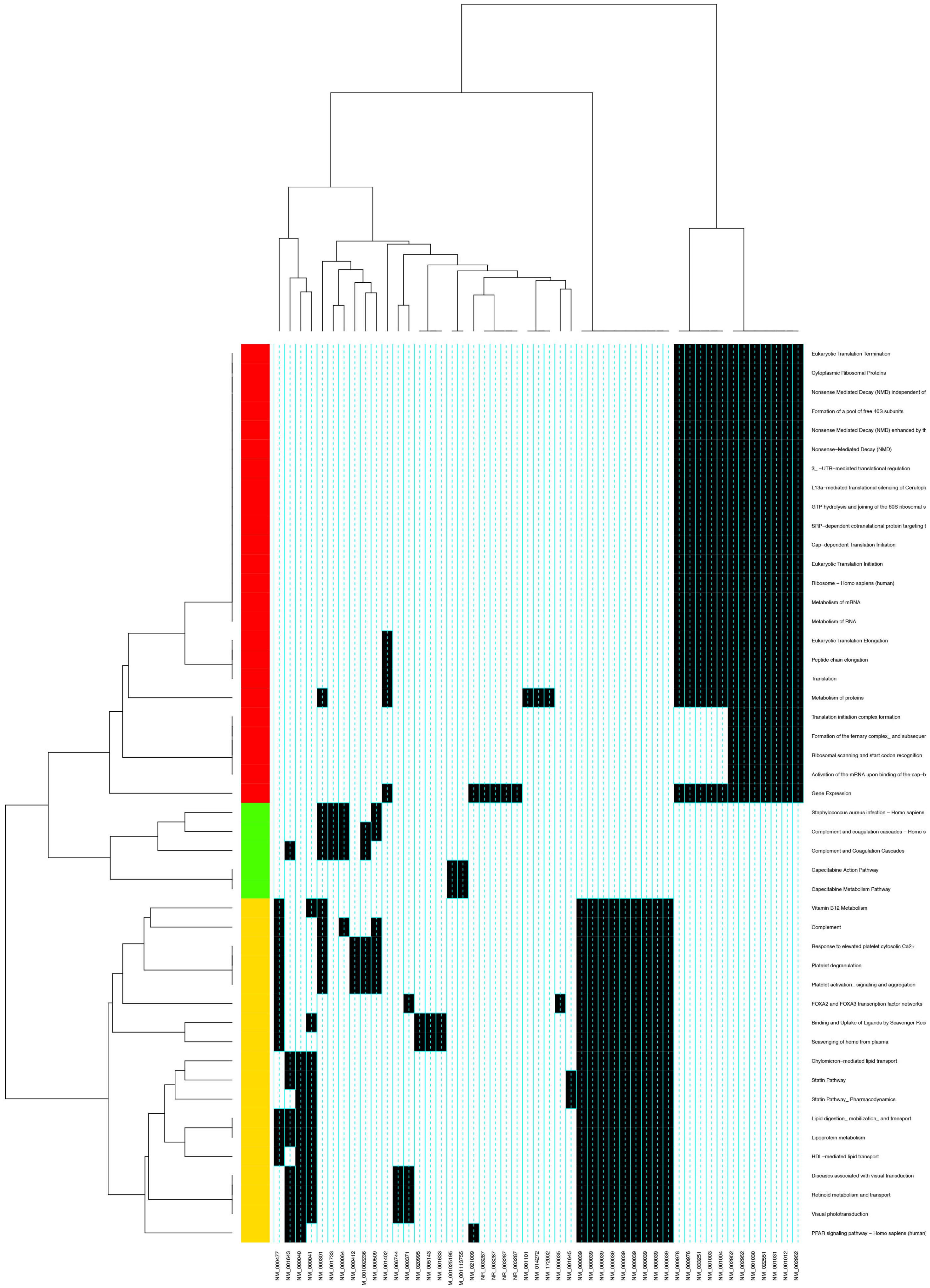
Eukaryotic Translation Termination
Cytoplasmic Ribosomal Proteins
Nonsense Mediated Decay (NMD) independent of
Formation of a pool of free 40S subunits
Nonsense Mediated Decay (NMD) enhanced by th
Nonsense–Mediated Decay (NMD)
3_ –UTR–mediated translational regulation
L13a–mediated translational silencing of Cerulopla
GTP hydrolysis and joining of the 60S ribosomal s
SRP–dependent cotranslational protein targeting t
Cap–dependent Translation Initiation
Eukaryotic Translation Initiation
Ribosome – Homo sapiens (human)
Metabolism of mRNA
Metabolism of RNA
Eukaryotic Translation Elongation
Peptide chain elongation
Translation
Metabolism of proteins
Translation initiation complex formation
Formation of the ternary complex_ and subsequen
Ribosomal scanning and start codon recognition
Activation of the mRNA upon binding of the cap–b
Gene Expression
Staphylococcus aureus infection – Homo sapiens
Complement and coagulation cascades – Homo s
Complement and Coagulation Cascades
Capecitabine Action Pathway
Capecitabine Metabolism Pathway
Vitamin B12 Metabolism
Complement
Response to elevated platelet cytosolic Ca2+
Platelet degranulation
Platelet activation_ signaling and aggregation
FOXA2 and FOXA3 transcription factor networks
Binding and Uptake of Ligands by Scavenger Rec
Scavenging of heme from plasma
Chylomicron–mediated lipid transport
Statin Pathway
Statin Pathway_ Pharmacodynamics
Lipid digestion_ mobilization_ and transport
Lipoprotein metabolism
HDL–mediated lipid transport
Diseases associated with visual transduction
Retinoid metabolism and transport
Visual phototransduction
PPAR signaling pathway – Homo sapiens (human)
NM_000477
NM_001643
NM_000040
NM_000041
NM_000301
NM_001733
NM_000064
NM_000412
M_001002236
NM_000509
NM_001402
NM_006744
NM_000371
NM_020995
NM_005143
NM_001633
M_001025195
M_001113755
NM_021009
NR_003287
NR_003287
NR_003287
NR_003287
NM_001101
NM_014272
NM_172002
NM_000035
NM_001645
NM_000039
NM_000039
NM_000039
NM_000039
NM_000039
NM_000039
NM_000039
NM_000039
NM_000039
NM_000976
NM_033251
NM_001003
NM_001004
NM_002952
NM_002952
NM_001030
NM_022551
NM_001031
NM_001012
NM_002952

Supplementary figure legend

Supplementary figure 1. Heatmap of compound

Horizontal line indicates ICC related compounds. Vertical line represents the pathways.

Supplementary figure 2. Heatmap of mRNA

Horizontal line indicates mRNAs related to ICC. Vertical line indicates pathways.

Supplementary methods

1 Data Normalization

1.1 mRNA/microRNA

"gProcessedSignal" was extracted from the microarray data and was normalized so as to have mean zero and standard deviation of one.

1.2 Compounds

No normalization was applied to the amount of compounds.

2 Principal Component Analysis and hierarchical clustering in order to choose PCs for feature extraction

Suppose expression matrix X is

$$(\vec{x}_1, \vec{x}_2, \cdots, \vec{x}_p)$$

where $\vec{x}_i$ is n dimensional vector. n is the number of mRNAs, miRNAs or componuds, and p is number of saples, i.e., 32. PCA allows to go from n points in a space of dimension $p(= 32)$ to n points in a space of dimension k (number of PCs considered). Each PC is a combination of p variables (p-dimensional vector); the number of PCs extracted is p. PCA was applied to matrix X.

`prcomp` function in [1] was used for principal component analysis and mRNAs, miRNAs and compounds were separately embedded into low dimensional space. Since there were 16 patients from which two samples (tumor and normal tissue) were extracted, in total 32 samples of mRNA, miRNA an compounds expression. Thus, there were up to 32 PCs for each of mRNA, miRNA and compounds expression. Each PCX ($X = 1, ..., 32$) should have weight CX_j^k from jth sample among 32 samples for kth expression (k stands for either mRNA, miRNA or compounds),

$$PCX_i^k = \sum_{j=1}^{32} CX_j^k x_{ij}$$

where PCX_i^k is PC score of ith probe (i.e., coordinate in the embedding space) of kth expression and $x_{ij}k$ is the expression of jth sample of ith probe. CX_j^k is also known as PC loadings. Negative signed absolute correlation coefficient $- \mid \rho_{X,X'}^{k,k'} \mid$ between $(CX_1^k, CX_2^k, ..., CX_{32}^k)$ and $(CX'^{k'}_1, CX'^{k'}_2, ..., CX'^{k'}_{32})$ were used as distance for hierarchical clustering. Hierarchical clustering was performed by `hclust` function in R[1] with `method="average"` option, thus it is Unweighted Pair Group Method using arithmetic Average (UPGMA).

3 PCA based unsupervised FE (PCAFE)

Using PCAFE [2, 3], mRNAs, miRNAs and compounds were extracted. mRNAs whose PC1 < -50, miRNAs whose $\sqrt{PC1^2 + PC2^2} > 10$, and compounds whose $\mid PC3 \mid > 0.1$ was extracted for further analysis.

4 Pathway analysis of obtained mRNAs, miRNAs, and compounds

In order to perform pathway enriched analyses, two servers were employed. For mRNAs and compounds, IMPaLA[4, 5] was used. For miRNAs, DIANA-mirpath[6, 7] with using Tarbase as target identification was used. For both servers, files including miRNAs (mature miRNA names), mRNAs (RefSeq mRNA) or compounds (KEGG compound ID) were uploaded. Enriched pathways were automatically extracted.

5 Discrimination between ICC, HCC and controls

As done before [2, 3], using extracted mRNAs, miRNAs, and compounds, we have tried to discriminate HCC, ICC and normal tissues (three class discrimination problem).

1. 32 samples are embedded into low dimensional space using either extracted mRNAs, miRNAs or compounds.

2. PC scores (i.e., coordinates in the embedded space) of each sample were used for discrimination.

3. Using PC scores up to optimal number of PCs, three classes were discriminated using linear discriminant analysis (LDA), where LDA was

performed using `lda` function in `MASS` package in R[1] with the options `CV=T, prior=rep(1/3,3)`.

4. Performance was evaluated by comparing true labels and `class` variables obtained by `lda` (`class` variables were obtained by leave one out cross validation since option `CV=T` was set).

6 Categorical regression performed for mRNAs, miRNAs and compounds in supplementary tables 2, 3, and 4

Categrical regression was performed to each of extracted mRNAs, miRNAs and compounds as

$$x_{ij} = C_i + C_{ICC}\delta_{j,ICC} + C_{ICC-NT}\delta_{j,ICC-NT} + C_{HCC}\delta_{j,HCC} + C_{HCC-NT}\delta_{j,HCC-NT}$$

and P-values associated with the regression analysis was provided. Here $\delta_{a,j}$ takes one if the j-th sample is equivalent to category a (a represents ICC, ICC-NT, HCC, or HCC-NT), otherwise it had a value of zero.

References

[1] R Core Team. *R: A Language and Environment for Statistical Computing*. R Foundation for Statistical Computing, Vienna, Austria, 2014.

[2] Y. Murakami, H. Toyoda, T. Tanahashi, J. Tanaka, T. Kumada, Y. Yoshioka, N. Kosaka, T. Ochiya, and Y. H. Taguchi. Comprehensive miRNA expression analysis in peripheral blood can diagnose liver disease. *PLoS ONE*, 7(10):e48366, 2012.

[3] Y. H. Taguchi and Y. Murakami. Principal component analysis based feature extraction approach to identify circulating microRNA biomarkers. *PLoS ONE*, 8(6):e66714, 2013.

[4] IMPaLA. http://impala.molgen.mpg.de/.

[5] A. Kamburov, R. Cavill, T. M. Ebbels, R. Herwig, and H. C. Keun. Integrated pathway-level analysis of transcriptomics and metabolomics data with IMPaLA. *Bioinformatics*, 27(20):2917–2918, Oct 2011.

[6] Diana-mirpath. http://diana.imis.athena-innovation.gr/DianaTools/index.php?r=mirpath/index.

[7] I. S. Vlachos, N. Kostoulas, T. Vergoulis, G. Georgakilas, M. Reczko, M. Maragkakis, M. D. Paraskevopoulou, K. Prionidis, T. Dalamagas, and A. G. Hatzigeorgiou. DIANA miRPath v.2.0: investigating the combinatorial effect of microRNAs in pathways. *Nucleic Acids Res.*, 40(Web Server issue):498–504, Jul 2012.
